# Modifiable Protective Strategies and System-Level Predictors of Professional Quality of Life in Oncology Nurses: A Secondary Analysis

**DOI:** 10.1177/23779608251407811

**Published:** 2025-12-22

**Authors:** Carolyn S. Phillips, Cheongin Rachel Im, Katie Trainum, Megan C. Thomas Hebdon

**Affiliations:** 1 12330 The University of Texas at Austin, School of Nursing, Austin, Texas, USA; 2 National Clinician Scholars Program, 6572 University of Pennsylvania, Philadelphia, Pennsylvania, USA; 3 16177 University of Utah, College of Nursing, Salt Lake City, Utah, USA

**Keywords:** Self-awareness, organizational culture, burnout, compassion fatigue, oncology nurses

## Abstract

**Introduction:**

Oncology nurses experience heightened risk for burnout and secondary traumatic stress (STS) due to sustained exposure to patient suffering, high mortality, and emotionally demanding care. These challenges are multifactorial, and limited research has examined modifiable protective factors or system-level predictors that support nurses’ well-being.

**Objective:**

To identify relationships among emotional well-being, protective strategies, and professional quality of life, and to examine modifiable predictors of burnout, STS, and compassion satisfaction in oncology nurses.

**Methods:**

This secondary analysis drew from baseline quantitative and qualitative data collected in two feasibility studies of the *Storytelling Through Music* intervention for oncology nurses recruited from U.S. inpatient and outpatient cancer centers (2018–2023). Quantitative measures included anxiety, depression, self-compassion, self-reflection and insight, and professional quality of life (burnout, STS, compassion satisfaction). Data were analyzed using descriptive statistics, correlations, and multiple regression. Qualitative data were analyzed using a descriptive approach to contextualize quantitative findings.

**Results:**

Among 67 oncology nurses (M age = 40 years, 96% female, 91% White), burnout correlated positively with anxiety and depression, and negatively with self-compassion, insight, and compassion satisfaction (all *p*'*s* < .001). Regression analyses showed burnout was predicted by lower self-compassion (*p* = .006), lower insight (*p* = .012), and greater perceived workplace support (*p* = .040; R^2^ = 0.42). Self-compassion predicted higher compassion satisfaction (*p* = .012). Qualitative findings contextualized these results, revealing limited workplace support and superficial self-care education as systemic barriers to oncology nurses’ well-being.

**Conclusions:**

Healthcare organizations and nursing schools must move beyond passive approaches to emotional support by embedding structured, skills-based interventions into practice and education. In clinical settings, this includes routine grief debriefings, accessible counseling, and psychologically safe spaces for peer and counseling support. Nursing curricula should embed experiential training in emotion regulation and self-compassion to prepare clinicians to manage grief, sustain empathy, and prevent burnout.

## Introduction

Oncology nurses face significantly elevated rates of anxiety, depression, burnout, suicide risk, and secondary traumatic stress (STS) due to the emotionally intense nature of their work and limited opportunities for emotional debriefing, which further exacerbate stress levels (Salehi et al., 2025; Shaqiqi et al., 2024). Studies show that oncology nurses experience higher levels of psychological distress compared to nurses in other specialties, with reported anxiety and depression rates ranging from 25% to 45% (De la Fuente-Solana et al., 2020; Yu et al., 2016). Burnout is also prevalent, with up to 50% of oncology nurses experiencing moderate to high levels, often linked to heavy workloads, emotional demands, and inadequate institutional support (Helaß & Maatouk, 2024) as well as intricate treatment protocols, high-risk medication administration, and frequent exposure to death, dying, bereavement, and family stress. Of great concern, nurses, in general, are at a higher risk for suicide than the general population, with female nurses showing a particularly elevated risk (Davidson et al., 2021; Nguyen et al., 2025).

High rates of burnout are not limited to oncology nurses in the United States. Across international studies, oncology nurses report high rates of burnout, with emotional exhaustion and depersonalization particularly elevated (Algamdi, 2022; Getie et al., 2025). In a study of oncology nurses in Spain, moderate to severe levels of anxiety, depression, and STS were documented (Arimon-Pagès et al., 2019). Further, they found that approximately half of oncology nurses met thresholds for high burnout or STS (Arimon-Pagès et al., 2019). A 2024 survey of oncology nurses in Oman reported that only 35% had high compassion satisfaction, while 65% and 60% of nurses had moderate levels of burnout and STS, respectively (Jacob et al., 2024). Taken together, international data underscore that oncology nurses across diverse settings are at high risk for burnout and STS and may struggle to maintain optimal professional quality of life. This prevalent issue is not confined to the U.S., highlighting a global need for supportive interventions.

## Review of Literature

Research indicates that predictors of burnout and STS in oncology nurses are multifactorial. Organizational contributors—such as excessive workload, inadequate staffing, and administrative burdens—drive emotional exhaustion and depersonalization, while personal factors—including cumulative grief from frequent patient deaths and ongoing moral distress—diminish resilience and amplify provider grief (Boyle & Steinheiser, 2021; Erami & Taghadosi, 2023; Musker & Othman, 2024; Yazdan et al., 2023). The emotional labor of oncology nurses can negatively affect their well-being, especially when self-awareness—the capacity to monitor and regulate one's internal states—is limited.

Kearney and Weininger's Self-Awareness-Based Model of Self-Care describes a dynamic, multidimensional process in which nurses cultivate presence and emotional attunement to themselves and others (Kearney & Weininger, 2011). Within the model, Kearney and Weininger propose that a clinician's level of self-awareness fundamentally shapes their response to occupational stress and patient suffering. The model illustrates two parallel pathways, determined by the clinician's capacity for self-awareness: one leading to negative consequences and the other to positive outcomes. When functioning with limited self-awareness, clinicians may lose perspective, experience heightened stress, and perceive empathy as burdensome, increasing their vulnerability to burnout and STS. In contrast, clinicians who cultivate greater self-awareness are more likely to maintain perspective, experience less stress and greater satisfaction, and engage in “exquisite empathy” that fosters healing connections with patients, colleagues, and themselves.

Emotion regulation skills are crucial for oncology nurses to remain emotionally balanced while caring for people with cancer. In addition to self-awareness, effective emotion regulation strategies such as cognitive reappraisal and stress reduction can help nurses manage work-related stress and emotions and improve both personal and professional well-being and quality of care (Duarte & Pinto-Gouveia, 2017b; Gross, 2015). Research suggests that nurses who employ adaptive emotion regulation techniques are less likely to experience compassion fatigue and more likely to exhibit resilience and emotional balance in their clinical practice (Delgado et al., 2017). Conversely, suppression of emotions, a maladaptive strategy, has been linked to increased psychological distress and decreased job satisfaction (Cybulska et al., 2022).

Self-awareness and self-compassion are modifiable emotion regulation strategies that can be taught to oncology nurses and can improve emotion regulation. Self-awareness allows nurses to recognize and understand their emotional responses to challenging clinical situations, thereby enabling more intentional and adaptive regulation strategies (Salem et al., 2025). Coupled with this, self-compassion, treating oneself with kindness and understanding during moments of distress, has been shown to buffer the negative psychological impacts of caregiving stress (Neff, 2003; Sinclair et al., 2017). Nurses who cultivate self-compassion are less likely to experience burnout and more likely to maintain emotional resilience, even in the face of patient suffering and loss (Durkin et al., 2016). These traits not only support the individual nurse's well-being but also enhance their capacity for empathy and sustained compassionate care.

Formal nursing education that intentionally incorporates self-care strategies, combined with institutional workplace support systems, can significantly enhance the well-being of nurses and foster sustained professional engagement. For instance, structured educational modules on stress management, mindfulness, boundary setting, and emotional regulation help nurses recognize early signs of compassion fatigue and implement self-care practices proactively (e.g., brief mindfulness breaks, reflective exercises) (Burner & Spadaro, 2023; Gifford & Kwasky, 2024; Higgins et al., 2025). When such individual skills are reinforced by organizational supports, such as protected time for wellness, mentoring, peer support groups, and a culture that normalizes self-care rather than stigmatizing it, nurses report greater emotional safety and less moral distress (Plummer et al., 2025). Together, formal education plus structural support creates a reinforcing “self-care environment” that helps preserve nurses’ psychological resources, reduce turnover, and ultimately improve both staff retention and quality of care.

The purpose of this study was to examine the relationships between emotional variables and modifiable protective strategies (self-compassion and self-awareness) and system-level factors (formal education about self-care and workplace support) as well as predictors of professional quality of life (burnout, STS, and compassion satisfaction) in oncology nurses. This study fills a gap in the literature by addressing both modifiable internal factors and organizational factors that impact professional quality of life.

## Methods

This study was a secondary analysis of baseline quantitative and qualitative data from two parent studies evaluating the feasibility and acceptability of the Storytelling Through Music intervention to help oncology nurses cope with work-related emotions. Unlike mixed-methods designs, multiple methods studies do not seek to integrate qualitative and quantitative data but rather to enhance the depth and rigor of findings through methodological triangulation (Morse, 2003). Ethics approval for the secondary analysis of de-identified baseline data was obtained from the University of Texas at Austin Institutional Review Board (STUDY0000583). All procedures adhered to the principles of the Declaration of Helsinki and relevant data protection regulations.

### Research Questions

This study aims to answer the following research questions: 1) What proportion of oncology nurses report access to workplace bereavement or emotional support, and how many received formal self-care education during their nursing training? 2) What are the relationships among emotional well-being (anxiety, depression), protective strategies (self-compassion, insight), and professional quality of life outcomes (burnout, STS, and compassion satisfaction)? 3) To what extent do modifiable protective strategies (self-compassion, insight) and system-level factors (formal self-care education, workplace support) predict professional quality of life outcomes—burnout, STS, and compassion satisfaction? 4) How do oncology nurses describe their experiences with workplace support for bereavement and formal education on self-care, and how do these perspectives contextualize quantitative findings?

### Participants and Sampling

Oncology nurse participants were recruited via convenience sampling from cancer care settings throughout the United States. Study 1 enrolled nurses in 2018, while Study 2 recruited between 2020–2023 (Phillips et al., 2020, 2021, 2024). Eligible participants were registered nurses practicing in inpatient and outpatient oncology units at National Cancer Institute-designated centers and community cancer centers. Exclusion criteria for the second study included past participants in a Storytelling Through Music study or program. Invitations were disseminated through professional nursing networks, institutional listservs, relevant social media forums, and word of mouth. All participants provided informed consent before baseline data collection.

### Data Collection

The baselined data collection included a background questionnaire to describe the sample (participants’ age, education, race, ethnicity, employment, number of years in nursing, number of years in oncology, highest level of nursing education, additional certifications, etc.) and a battery of validated self-report measures emotional well-being (anxiety and depression), protective factors (self-reflection and insight, self-compassion), and professional quality of life (burnout, STS, and compassion satisfaction). Additionally, each participant was asked whether their workplace offers bereavement or emotional support, and if they had received formal self-care education during their formal nursing education. Free-text responses were also collected to allow participants to describe their experiences with workplace bereavement support and education on self-care. All the quantitative and qualitative data were collected in one survey (Supplemental File 1).

### Outcome Measures

**Emotional Well-being.** Anxiety and depression were measured with the Patient-Reported Outcomes Measurement Information System Anxiety Short Form (six items) and Depression Short Form (eight items), respectively (Cella et al., 2010). Higher scores indicate higher amounts of the constructs. Both scales had high internal consistency reliability scores in this study—Cronbach's alpha for anxiety was 0.914, and 0.919 for depression.

**Protective Factors.** Self-awareness was operationalized using the Self-Reflection and Insight Scale (Grant et al., 2002), which assesses self-reflection engagement (6 items), self-reflection need (6 items), and insight (8 items). Higher scores indicate higher amounts of the construct. Reliability in this study was 0.851, 0.846, and 0.746, respectively. Self-compassion was measured with the Self-Compassion Scale (26 items) (Neff, 2003). A higher score indicates a greater degree of the construct. The scale demonstrates good reliability and discriminant validity. In this study, the reliability was 0.965.

**Professional Well-being.** The Professional Quality of Life Scale is a 30-item, self-report measure that yields three discrete scales: burnout (feelings of hopelessness and difficulties in dealing with work effectively), STS (one's work-related, secondary exposure to highly stressful events), and compassion satisfaction (the pleasure derived from doing one's work well) (Stamm, 2010). For this study, internal consistency was 0.784 for burnout, 0.782 for STS, and 0.901 for compassion satisfaction.

### Data Analysis

For the quantitative component, all data management and statistical analyses were performed using the R Studio statistical program. For the missing values, multiple imputations by chained equations were conducted. Descriptive and frequency statistics were calculated to summarize the variables included in the analyses. Because this was a secondary analysis of baseline data drawn from two feasibility studies, no a priori power analysis was conducted for sample estimation. The primary aim of the parent studies was to assess the feasibility and acceptability of the Storytelling Through Music intervention rather than to test statistical hypotheses. For the present secondary analysis, which employed multiple linear regression with four predictor variables, post hoc estimation of sample adequacy revealed a minimum sample size of 82 would be required to detect small-to-moderate effect sizes with adequate power (0.80, α = .05). Thus, while the current sample of 67 was slightly underpowered for small effects, it was sufficient to explore moderate-to-large effects and to generate preliminary evidence to inform future, fully powered studies.

Pearson correlations were used to identify the association between anxiety, depression, self-reflection and insight, self-compassion, burnout, STS, and compassion satisfaction. Multiple linear regression was conducted to analyze the influence of predictors (educational support, workplace support, insight, and self-compassion) on burnout, STS, and compassion satisfaction. Anxiety and depression were excluded from the regression models to prevent conceptual overlap with the outcome variables and to maintain focus on modifiable predictors of professional quality of life. Because anxiety and depression are established outcomes of occupational stress rather than antecedent factors, including them could confound the interpretation of protective mechanisms. Instead, the analysis centered on theoretically driven and actionable constructs (self-compassion, self-reflection and insight, workplace support, and formal education about self-care) that can be targeted through intervention and organizational strategies.

Open-ended survey responses were analyzed using a qualitative descriptive approach, an appropriate method for capturing participants’ perspectives in everyday language and staying close to their expressed meanings (Sandelowski, 2000). Because the qualitative data consisted of brief written comments rather than full narratives or interviews, this approach prioritized surface-level description over deep interpretation, which contextualizes the quantitative findings. Responses were organized according to the two guiding prompts related to workplace support and formal education about self-care. Within each category, text was read multiple times for familiarity, and meaning units were identified and coded inductively to reflect key patterns in participants’ experiences. Two researchers independently reviewed the coded data and discussed discrepancies until consensus was achieved, enhancing credibility and confirmability. Standards for reporting cross-sectional and qualitative research guidelines were followed (Tong et al., 2007; von Elm et al., 2007).

## Results

### Participant Characteristics

Overall, 99 participants were screened during the parent studies, and 67 participants were enrolled and included in this secondary analysis. Table 1 summarizes the demographic characteristics of the study sample. The average age of participants was 40.0 years (SD = 11.5). Most participants identified as female (95.5%) and White (91.0%), with 82.1% identifying as non-Hispanic. Table 2 presents occupational characteristics. Participants reported an average of 13.1 years (SD = 10.6) of nursing experience, with most holding a bachelor's degree (64.2%) and working in outpatient settings (79.1%). The oncology nurses primarily worked with adults (85.1%), and 49.3% worked in medical oncology specialties.

**Table 1.** Background Characteristics.

	Overall (N = 67)
Age	
Mean (SD)	40.0 (11.5)
Median [Min, Max]	37.0 [24.0, 69.0]
Gender	
Female	64 (95.5%)
Male	3 (4.5%)
Race	
Asian	1 (1.5%)
Black/AA	1 (1.5%)
NH/PI	1 (1.5%)
White	61 (91.0%)
Mixed	2 (3.0%)
Ethnicity	
Hispanic	12 (17.9%)
Not Hispanic	55 (82.1%)
Marital status	
Single	16 (23.9%)
Married/Partnered	42 (62.7%)
Divorced	8 (11.9%)
Widowed	1 (1.5%)

**Table 2.** Occupational Characteristics.

	Overall (N = 67)
RN education	
Associate	8 (11.9%)
Bachelor	43 (64.2%)
Graduate	16 (23.9%)
RN experience	
Mean (SD)	13.1 (10.6)
Median [Min, Max]	10.0 [1.50, 51.0]
Employment status	
Full-time	57 (85.1%)
Part-time	7 (10.4%)
Per diem	3 (4.5%)
Practice setting	
Inpatient	10 (14.9%)
Outpatient	53 (79.1%)
Other	4 (6.0%)
Patient population	
Adult	57 (85.1%)
Pediatric	8 (11.9%)
Both	1 (1.5%)
Specialty	
Hemo/Onc/BMT	12 (17.9%)
MedOnc	33 (49.3%)
RadOnc	4 (6.0%)
Combination	4 (6.0%)
Other	13 (19.4%)
Certifications	
Yes	28 (41.8%)
No	39 (58.2%)
Formal self-care education	
Yes	23 (34.3%)
No	44 (65.7%)
Self-care continuing education (CEUs)	
Yes	12 (17.9%)
No	55 (82.1%)
Workplace support	
Yes	23 (34.3%)
No	43 (64.2%)

### Correlations Between Emotional Well-Being, Protective Factors, and Professional Quality of Life

Pearson correlation analyses revealed several significant associations among the key study variables, with many demonstrating moderate to strong effect sizes (Table 3). The strongest correlation was a positive association between anxiety and depression (r = 0.67), and both were positively associated with burnout (anxiety: r = 0.47, depression: r = 0.54). Anxiety was also negatively correlated with insight (r = –0.42) and self-compassion (r = –0.53). Similarly, depression was negatively associated with insight (r = –0.39), self-compassion (r = –0.58), and compassion satisfaction (r = –0.39). Self-compassion and compassion satisfaction were strongly positively correlated (r = 0.47). Insight and self-compassion also showed a strong positive correlation (r = 0.60), and both were positively associated with compassion satisfaction (r = 0.43 and 0.47, respectively). Moreover, burnout was strongly negatively associated with insight (r = –0.57), self-compassion (r = –0.57), and compassion satisfaction (r = –0.63). STS was positively associated with anxiety (r = 0.42), depression (r = 0.32), and burnout (r = 0.44). In contrast, it was negatively associated with insight (r = -0.36) and self-compassion (r = –0.25).

**Table 3.** Correlations Between Study Variables.

Variables	Mean	SD	Range	1	2	3	4	5	6	7	8	9	10	11
1-Age (years)	40.03	11.45	24–69 *^a^*	1.00										
2-Nursing Experience (years)	13.15	10.57	1.5–51 *^a^*	0.86***	1.00									
3-Anxiety	12.76	5.02	6–30 *^b^*	-0.06	-0.20	1.00								
4-Depression	14.55	5.30	8–40 *^b^*	0.08	-0.05	0.67***	1.00							
5-Self-reflection (engagement)	24.85	5.38	6–36 *^b^*	0.16	0.17	0.04	-0.06	1.00						
6-Self-reflection (need)	27.07	4.87	6–36 *^b^*	-0.06	-0.10	0.36**	0.21	0.58***	1.00					
7-Insight	34.16	4.83	8–48 *^b^*	0.24*	0.30*	-0.42***	-0.39**	0.30*	-0.25*	1.00				
8-Self-compassion	82.33	20.09	26–130 *^b^*	0.18	0.24	-0.53***	-0.58***	0.12	-0.19	0.60***	1.00			
9-Burnout	23.13	5.39	10–50 *^b^*	-0.07	-0.17	0.47***	0.54***	-0.19	0.23	-0.57***	-0.57***	1.00		
10-Secondary Traumatic Stress	22.76	5.67	10–50 *^b^*	0.07	0.02	0.43***	0.32**	0.03	0.21	-0.36**	-0.25*	0.44***	1.00	
11-Compassion Satisfaction	40.67	5.78	10–50 *^b^*	0.24*	0.30*	-0.25*	-0.39**	0.30*	0.06	0.43***	0.47***	-0.63***	-0.13	1.00

* p *<* 0.05, ** p *<* 0.01, ***p *<* *0.001.*

*^a^Actual range*, *^b^Instrument range.*

### Regression Analysis

Multiple regression analysis was used to test models for modifiable protective strategies and system-level factors that may predict professional quality of life (burnout, STS, and compassion satisfaction) (Table 4). Each of the predictor variables had a significant bivariate correlation with either burnout, compassion fatigue, or compassion satisfaction.

**Table 4.** Regression Models.

	Burnout first model^a^				Secondary traumatic stress second model^b^				Compassion satisfaction third model^c^			
Variables	B	S.E.	p-Value	CI	B	S.E.	p-Value	CI	B	S.E.	p-Value	CI
(Constant)	38.48	4.64	<.001	29.21–47.76	39.70	5.99	<.001	27.72–51.69	26.01	5.66	< .001	14.69–37.32
Self-care education	0.45	1.12	.692	−1.80–2.69	−2.45	1.45	.097	−5.35–0.46	1.81	1.37	.192	−0.93–4.55
Work support	2.36	1.12	.040	0.11–4.61	0.12	1.45	.933	−2.78–3.03	−1.75	1.37	.208	−4.49–1.00
Insight	−0.36	0.14	.012	−0.64 - −0.08	−0.31	0.18	.097	−0.67–0.06	0.18	0.17	.293	−0.16–0.52
Self-compassion	−0.09	0.03	.006	−0.16 - −0.03	−0.03	0.04	.446	−0.12–0.05	0.10	0.04	.012	0.02–0.18

**Notes:** ^a^Residual standard error: 4.151 on 61 degrees of freedom (1 observation deleted due to missingness) Multiple R-squared: 0.4524, Adjusted R-squared: 0.4165 F-statistic: 12.597 on 4 and NA DF, p-value: *1.56e-07*; ^b^Residual standard error: 5.362 on 61 degrees of freedom (1 observation deleted due to missingness) Multiple R-squared: 0.1735, Adjusted R-squared: 0.1193 F-statistic: 3.201 on 4 and NA DF, p-value: *0.0188*; ^c^Residual standard error: 5.062 on 61 degrees of freedom (1 observation deleted due to missingness) Multiple R-squared: 0.2818, Adjusted R-squared: 0.2347 F-statistic: 5.984 on 4 and NA DF, p-value: *0.000396.*

**Predictors of Burnout***.* The overall model was significant (F(4, 61) = 12.60, p < 0.001), explaining approximately 41.7% of the variance in burnout scores (R² = 0.452, adjusted R² = 0.417). Among the predictors, work support (β = 2.36, p = 0.040) and insight (β = –0.36, p = 0.012) were statistically significant. Additionally, self-compassion was a significant predictor (β = –0.09, p = 0.006). Self-care education was not a significant predictor (β = 0.45, p = 0.692).

**Predictors of STS***.* The overall model was statistically significant (F(4, 61) = 3.20, p = 0.019), explaining 11.9% of the variance in STS scores (R² = 0.174, adjusted R² = 0.119). However, none of the predictors reached statistical significance. Insight (β = –0.31, p = 0.097) and self-care education (β = –2.45, p = 0.097) approached significance. Self-compassion (β = –0.03, p = 0.446) and work support (β = 0.12, p = 0.933) were not statistically associated with STS.

**Predictors of Compassion Satisfaction***.* The regression model for compassion satisfaction was also statistically significant (F(4, 61) = 5.98, p < .001), accounting for approximately 23.5% of the variance in compassion satisfaction scores (R² = 0.282, adjusted R² = 0.235). Among the predictors, self-compassion emerged as a significant positive predictor (β = 0.10, p = 0.012). Other predictors, including insight (β = 0.18, p = 0.293), self-care education (β = 1.81, p = 0.192), and work support (β = –1.75, p = 0.208), were not statistically significant.

### Qualitative Results

Analysis of participants’ qualitative comments revealed two overarching themes: 1) Limited and Inaccessible Workplace Support, and 2) Minimal and Superficial Education on Self-Care, that together illustrate systemic gaps in institutional and educational preparation to manage professional grief and sustain emotional well-being.

### Theme 1: Limited and Inaccessible Workplace Support

Only one-third (34%) of participants perceived that their institutions provided workplace support for bereavement and work-related emotions. Across settings, nurses described the absence or inadequacy of formal support mechanisms, characterizing them as “rudimentary” or “passive.” Most participants reported that acknowledgment of loss was limited to automated or administrative gestures, such as email notifications following a patient's death, with no structured opportunities for follow-up, reflection, or debriefing. While some organizations offered Employee Assistance Programs (EAPs), on-site social workers, or manager-led debriefings, participants emphasized that these resources were difficult to access due to conflicting work schedules, staffing shortages, or being based at off-site satellite clinics. As one nurse explained, “All programs are held at [the primary location], and because I work different satellites, it does not allow us to attend most of the programs.” Even when support was available, several nurses expressed a desire for connection “from within” their teams rather than relying on external hotlines or services: “I do not feel we are supported in compassion for ourselves and just overall self-care. Yes, I can call the EAP hotline; however, I would feel better if it came from inside.” In the absence of formal programs, some participants described creating informal networks—such as message boards or peer check-ins—to process grief and support one another emotionally.

### Theme 2: Minimal and Superficial Education on Self-Care

Similarly, only 34% of participants reported receiving formal education about self-care. Participants who had received formal education about self-care in nursing school stated that it was cursory, with general admonitions rather than hands-on skill development. They described receiving general encouragements, such as “self-care prevents burnout,” or general encouragements like “Listening to my body and seeing a doctor when something feels off,” but often without corresponding instruction in practical skills or reflective practices. For example, one nurse noted, “Professors said how important it is to take care of yourself but did not teach specific techniques.” Others recounted isolated positive experiences, such as structured reflection or debriefing during senior practicums, that provided meaningful opportunities to integrate self-care into professional practice. A few practical lessons emerged, such as “During my senior practicum, I quickly learned the importance of scheduling time for personal reflection, and debriefing is still super helpful for my self-care.” Despite these exceptions, most participants emphasized a lack of depth and retention of self-care education, noting that the content was seldom reinforced once they entered clinical practice. Collectively, these findings highlight a critical gap between the acknowledgment of self-care as a professional value and the provision of concrete, sustainable strategies to enact it in the emotionally demanding context of nursing.

## Discussion

This study examined modifiable protective strategies and system-level predictors of professional well-being—specifically burnout, STS, and compassion satisfaction—among U.S. oncology nurses. Notably, most of the participants worked in the outpatient setting. Outpatient oncology nurses experience unique emotional and logistical stressors, balancing high patient volumes and complex coordination demands with the emotional labor of sustaining long-term relationships through repeated loss and grief (Ko & Kiser-Larson, 2016). Compared with inpatient oncology nurses, who often manage acute crises, outpatient nurses face the chronic emotional toll of witnessing patients’ prolonged treatment and decline, underscoring the need for workplace strategies such as structured grief debriefings, counseling access, and protected recovery time (Ko & Kiser-Larson, 2016).

Of the protective strategies examined, higher levels of self-compassion and personal insight (a component of self-awareness) were significantly predictive of lower levels of burnout, reinforcing Kearney and Weininger's Self-Awareness-Based Model of Self-Care. Nurses with greater self-compassion are less prone to psychological distress and more resilient in the face of loss and suffering (Durkin et al., 2016; Sinclair et al., 2017). In a mixed-methods study of hospital nurses, self-compassion had a moderating effect on compassion fatigue and even showed a predictive (protective) relationship—nurses scoring higher in self-compassion were less likely to experience severe STS (Upton, 2018). Nurses who practice self-kindness and mindfulness toward their own struggles tend to report less emotional exhaustion and greater well-being (Sharma et al., 2025). Mindfulness-based interventions have demonstrated efficacy in reducing compassion fatigue, burnout, and stress, while also increasing nurses’ life satisfaction, mindfulness, and self-compassion (Duarte & Pinto-Gouveia, 2016, 2017a). These findings suggest that cultivating mindfulness and present-moment coping skills can bolster resilience in high-stress oncology environments.

In addition to being a significant predictor of burnout, insight was marginally significant in predicting STS. Insight offers protection against burnout and STS by enabling nurses to recognize early signs of emotional depletion and proactively engage in self-care behaviors. In this study, insight and self-compassion were found to be strongly correlated, highlighting an opportunity to develop synergistic interventions that teach nurses how to recognize internal states while extending kindness toward themselves. Targeting these modifiable traits through structured interventions, such as reflective practices, mindfulness training, and self-compassion exercises, can improve emotional regulation, well-being, and care quality (Delgado et al., 2017; Finlay-Jones et al., 2015).

Of the system-level predictors, self-care education in nursing school neared significance as a predictor of STS. Moreover, and surprisingly, although workplace support is typically assumed to be protective, greater perceived support was unexpectedly associated with higher levels of burnout in this sample. This may reflect reactive help-seeking among more distressed nurses or variability in the quality and accessibility of support offered. Notably, most participants reported “no” to items asking whether they had previously received workplace support or continuing education on self-care, revealing a gap in institutional resources.

In the qualitative data, the nurses described most existing programs as rudimentary or inaccessible. In the absence of consistent institutional support, many participants relied on informal peer outlets for grief processing. The limited availability and perceived ineffectiveness of institutional supports are concerning. Structural barriers, such as staffing shortages, geographic distance from central campuses, and limited services offered outside of the 9–5 workday, further restrict access. These findings echo earlier reports that many nurses must self-navigate emotional recovery or depend on informal peer support (Cunningham & Geyer, 2023; Phillips et al., 2020).

Workplace environments that are characterized by open communication and mental health resources are associated with lower levels of stress and burnout among nurses (Kelly et al., 2021). Furthermore, workplace initiatives that include training in emotional intelligence, mindfulness, and coping strategies have also demonstrated benefits in enhancing resilience and emotional regulation (Cohen et al., 2023; Medland et al., 2004). Despite this evidence, many healthcare institutions lack formal, ongoing interventions to address emotional well-being, and such resources are often reactive rather than preventive (Melnyk et al., 2025). In addition, time constraints, staffing shortages, and a culture of emotional suppression in nursing can hinder access to and utilization of available support (Kirk, 2024; McDermott et al., 2021; Wells, 2024). The gap between evidence and practice highlights the need for the integration of systemic, evidence-based emotional support programs into both nursing education and workplace policies.

Transforming healthcare culture to prioritize clinician wellness requires a systemic shift from reactive burnout mitigation to proactive support for professional fulfillment. The Stanford Professional Fulfillment Model emphasizes three interrelated drivers of well-being: culture of wellness, efficiency of practice, and personal resilience (Shanafelt, n.d.). Within this framework, fostering a culture of wellness involves an institutional commitment to psychological safety, transparent communication, and shared values that support both patient care and clinician well-being. For oncology nurses who routinely face emotionally taxing situations, a wellness-oriented culture would include structured emotional support, visible leadership investment in mental health, and integration of wellness metrics into organizational performance reviews. Rather than placing the burden solely on individuals to be resilient in a sick system, this model underscores the role of system-level changes that align professional values with daily practice. Thus, addressing burnout requires not only individual-level interventions but also organizational commitment—improving nurse-to-patient ratios, ensuring manageable workloads, promoting work–life balance, and establishing a supportive infrastructure for emotional well-being.

### Strengths and Limitations

There are two notable strengths to this study: 1) its multiple methods design, which provided nuanced insights into the phenomena of burnout and STS among oncology nurses, and 2) the use of a national sample across diverse cancer centers, which enhances the representativeness and relevance of the findings. However, several limitations should be noted. The cross-sectional design prevents causal inferences. Although the sample size was adequate for detecting moderate-to-large effects, the study was not powered to detect small effect sizes; therefore, findings should be interpreted as preliminary and hypothesis-generating rather than confirmatory. Third, this was a secondary analysis of feasibility-study data may have introduced selection bias, as participants were already engaged in a self-care intervention. The use of convenience sampling and a predominantly White female sample limits generalizability to more diverse nursing populations. Last, most participants worked in outpatient settings, which may limit the applicability of findings to inpatient or other clinical environments. These factors should be considered when interpreting the findings and applying them to broader nursing populations.

### Implications for Practice and Education

The study results suggest that multi-level interventions are required. Healthcare organizations should establish and promote accessible, protected-time bereavement support—such as on-unit debriefings and peer-facilitated grief circles—to normalize emotional processing and reduce the support–access gap. Leadership should model emotional openness and reinforce the legitimacy of seeking support. Regularly scheduled, team-based debriefings and protected time for reflection can help mitigate cumulative stress and promote shared resilience. Embedding support within the workflow rather than as an optional add-on may improve access and effectiveness.

Concurrently, nursing educators must incorporate competency-based self-awareness and self-care modules into foundational curricula, ensuring that graduates possess both the theoretical understanding and practical skills to safeguard their emotional well-being. Leadership at all levels should champion these initiatives as essential components of workforce sustainability and patient safety. Many participants noted a disconnect between broad encouragements and the absence of concrete, skill-based instruction—highlighting a missed opportunity to equip future nurses with practical tools for emotional resilience. Curricula should incorporate experiential learning on self-awareness, self-compassion, stress management, and emotion regulation. Nursing faculty must be equipped to model and teach these skills, ensuring students are not only exposed to principles of self-care but are also taught how to do it meaningfully and sustainably.

#### Future Directions and Research Implications

Interventions that explicitly cultivate self-compassion, psychological insight, and reflective capacity may mitigate burnout and STS while strengthening compassion satisfaction. Equally important is the need to evaluate system-level strategies, such as structured debriefings, grief literacy education, and embedded peer support programs, that normalize emotional processing within oncology care teams. Longitudinal and mixed-methods designs are warranted to examine how shifts in these resources influence well-being trajectories over time and across diverse oncology settings. Finally, intervention studies that integrate emotional intelligence and self-care curricula into undergraduate and continuing nursing education could provide sustainable models for preventing burnout and fostering professional fulfillment throughout the nursing career lifespan.

## Conclusion

This study highlights the importance of both individual and systemic approaches in supporting the emotional well-being of oncology nurses. Self-compassion and personal insight emerged as protective factors against burnout and contributors to compassion satisfaction, pointing to the value of interventions that cultivate emotional awareness. At the same time, findings revealed gaps in institutional support, with many nurses reporting limited access to effective workplace resources for emotional processing and grief support. These results underscore the need for healthcare organizations to integrate structured, evidence-based emotional support into their practices and education. By fostering a culture of wellness that aligns with the realities of oncology care, institutions can promote resilience, reduce burnout, and sustain the capacity for compassionate caregiving across the nursing workforce.

## Supplemental Material

sj-pdf-1-son-10.1177_23779608251407811 - Supplemental material for Modifiable Protective Strategies and System-Level Predictors of Professional Quality of Life in Oncology Nurses: A Secondary Analysis Supplemental material, sj-pdf-1-son-10.1177_23779608251407811 for Modifiable Protective Strategies and System-Level Predictors of Professional Quality of Life in Oncology Nurses: A Secondary Analysis by Carolyn S. Phillips, Cheongin Rachel Im, Katie Trainum and Megan C. Thomas Hebdon in SAGE Open Nursing

sj-docx-2-son-10.1177_23779608251407811 - Supplemental material for Modifiable Protective Strategies and System-Level Predictors of Professional Quality of Life in Oncology Nurses: A Secondary Analysis Supplemental material, sj-docx-2-son-10.1177_23779608251407811 for Modifiable Protective Strategies and System-Level Predictors of Professional Quality of Life in Oncology Nurses: A Secondary Analysis by Carolyn S. Phillips, Cheongin Rachel Im, Katie Trainum and Megan C. Thomas Hebdon in SAGE Open Nursing

sj-docx-3-son-10.1177_23779608251407811 - Supplemental material for Modifiable Protective Strategies and System-Level Predictors of Professional Quality of Life in Oncology Nurses: A Secondary Analysis Supplemental material, sj-docx-3-son-10.1177_23779608251407811 for Modifiable Protective Strategies and System-Level Predictors of Professional Quality of Life in Oncology Nurses: A Secondary Analysis by Carolyn S. Phillips, Cheongin Rachel Im, Katie Trainum and Megan C. Thomas Hebdon in SAGE Open Nursing
