# Supplementary material for: Modifiable Protective Strategies and System-Level Predictors of Professional Quality of Life in Oncology Nurses: A Secondary Analysis
Source: SAGE Open Nurs. 2025 Dec 22;11:23779608251407811. doi: 10.1177/23779608251407811 (PMC12722642; doi:10.1177/23779608251407811)
Supplement: sj-pdf-1-son-10.1177_23779608251407811 - Supplemental material for Modifiable Protective Strategies and System-Level Predictors of Professional Quality of Life in Oncology Nurses: A Secondary Analysis [file sj-pdf-1-son-10.1177_23779608251407811.pdf]

Appendix B: Questionnaires and Survey Instruments

Background Information
(Timing: T1)

1. What is your preferred method of communication? (please check your #1 preference but list all options)

    ☐ Text: ____

    ☐ Email: ____

    ☐ Phone: ____

2. What is your age? ____

3. Gender identification?
    ☐ Female
    ☐ Male
    ☐ Transgender
    ☐ Gender variant/non-conforming
    ☐ Not listed (specify)
    ☐ Prefer not to answer

4. What is your race?
    ☐ Asian
    ☐ Black or African-American
    ☐ Native Hawaiian or other Pacific Islander
    ☐ Native American or Alaska Native
    ☐ White
    ☐ Mixed race
    ☐ Prefer not to answer

5. What is your ethnicity?
    ☐ Hispanic or Latino
    ☐ Not Hispanic or Latino
    ☐ Prefer not to answer

6. What is your marital status?
    ☐ Single
    ☐ Partnered/Married
    ☐ Divorced
    ☐ Widowed

7. Do you consider yourself religious?
    ☐ Yes – If applicable, please specify your religion and/or denomination:________________________
    ____
    ____
    ____
    ☐ No

8. Do you consider yourself spiritual?
    ☐ Yes
    ☐ No

9. Highest level of nursing education?
   - ☐ Associate's Degree
   - ☐ Bachelor's Degree
   - ☐ Diploma
   - ☐ DNP
   - ☐ EdD
   - ☐ Master's Degree
   - ☐ Other (specify)
   - ☐ PhD/DNSc Degree

10. Highest level of other education?
    - ☐ Associate's Degree
    - ☐ Bachelor's Degree
    - ☐ Master's Degree
    - ☐ Doctoral Degree

11. Years of Nursing Experience? ____

12. Employment status?
    - ☐ Full-time
    - ☐ Part-time
    - ☐ Per diem
    - ☐ Retired
    - ☐ Unemployed

13. What is your current practice setting?
    - ☐ Inpatient
    - ☐ Ambulatory
    - ☐ Other

14. What is your primary patient setting? (Check all that apply)
    - ☐ Adult
    - ☐ Pediatric
    - ☐ Older adult
    - ☐ N/A

15. What is your practice area? ____

16. What certifications do you hold? ____

17. What drew you to oncology nursing as a career?

    ____
    ____
    ____
    ____
    ____

18. Have you had any recent family deaths during the pandemic? Do not have to be COVID-related.

    - ☐ Yes – Please explain:____
      ____
      ____
      ____
    - ☐ No

19. Have you had any family members die of cancer in the past? If so,

☐ Yes – Who and how old where you?____
____
____
____

☐ No

20. Have you had a sibling or parent diagnosed with cancer?

☐ Yes – Who and how old where you?____
____
____
____

☐ No

21. Did your nursing education teach you how to care for yourself while caring for others?

☐ Yes – Please explain:____
____
____
____

☐ No

22. Have you attended any CE programs about self-care for the caregiver?

☐ Yes – Please describe:____
____
____
____

☐ No

23. Do you feel your workplace has sufficient staff support and/or programs in place to effectively deal with work-related emotions?

☐ Yes - Please explain: ____
____
____
____

☐ No - Please explain:____
____
____

24. What has been your primary nursing role during the pandemic? ______________________

25. Please tell me how your mental health has been affected during the pandemic?

____
____
____
____

26. Do you intend to leave your job within the next 6 months?

☐ Yes

☐ No

27. Do you intend to leave nursing because of the pandemic?

☐ Yes

☐ No

28. Do you have a regular writing practice?
    - ☐ Yes
    - ☐ No

29. Do you sing or play a musical instrument?
    - ☐ Yes
    - ☐ No

30. What kind of music do you prefer? Select all that apply
    - ☐ Hip Hop
    - ☐ Country
    - ☐ Rap
    - ☐ Folk
    - ☐ Americana
    - ☐ Jazz
    - ☐ Classical
    - ☐ I don’t have a preference
    - ☐ Other

Data Collection 1-Item Questionnaire

(Timing: T2, T3, T4)

1. Have you had a change in your job situation since starting this program?
   ☐ Yes; Please explain:____
   ____
   ____
   ____

   ☐ No

SELF-REPORT INSTRUMENTS

(Timing: T1, T2, T3, T4)

1. Anxiety Short Form 6a – PROMIS Item Bank v1.0
2. Depression Short Form 8b – PROMIS Item Bank v1.0
3. Self-Reflection and Insight Scale
4. Self-Compassion Scale
5. Professional Quality of Life

Anxiety Short Form 6a – PROMIS Item Bank v1.0

Please respond to each question or statement by marking one box per row.

In the past 7 days…

STATEMENTS	Never	Rarely	Sometimes	Often	Always
1. I felt fearful	1	2	3	4	5
2. I found it hard to focus on anything other than my anxiety	1	2	3	4	5
3. My worries overwhelmed me	1	2	3	4	5
4. I felt uneasy	1	2	3	4	5
5. I felt nervous	1	2	3	4	5
6. I felt like I needed help for my anxiety	1	2	3	4	5

Depression Short Form 8b – PROMIS Item Bank v1.0

Please respond to each question or statement by marking one box per row.

In the past 7 days…

STATEMENTS	Never	Rarely	Sometimes	Often	Always
1. I felt worthless	1	2	3	4	5
2. I felt that I had nothing to look forward to	1	2	3	4	5
3. I felt helpless	1	2	3	4	5
4. I felt sad	1	2	3	4	5
5. I felt like a failure	1	2	3	4	5
6. I felt depressed	1	2	3	4	5
7. I felt unhappy	1	2	3	4	5
8. I felt hopeless	1	2	3	4	5

Self-Reflection and Insight Scale

Please read the following questions and circle the response that indicates the degree to which you agree or disagree with each statement. Try to be accurate, but work quite quickly. Do not spend too much time on any question.

STATEMENTS	Disagree Strongly	Disagree	Disagree Slightly	Agree Slightly	Agree	Agree Strongly
1. I don't often think about my thoughts (R)	1	2	3	4	5	6
2. I am not really interested in analyzing my behavior (R)	1	2	3	4	5	6
3. I am usually aware of my thoughts	1	2	3	4	5	6
4. I am often confused about the way that I really feel about things (R)	1	2	3	4	5	6
5. It is important for me to evaluate the things that I do	1	2	3	4	5	6
6. I usually have a very clear idea about why I have behaved in a certain way	1	2	3	4	5	6
7. I am very interested in examining what I think about	1	2	3	4	5	6
8. I rarely spend time in self-reflection (R)	1	2	3	4	5	6
9. I'm often aware that I am having a feeling, but I often don't quite know what it is (R)	1	2	3	4	5	6
10. I frequently examine my feelings	1	2	3	4	5	6
11. My behavior often puzzles me (R)	1	2	3	4	5	6
12. It is important to me to try to understand what my feelings mean	1	2	3	4	5	6
13. I don't really think about why I behave in the way that I do (R)	1	2	3	4	5	6
14. Thinking about my thoughts makes me more confused (R)	1	2	3	4	5	6
15. I have a definite need to understand the way my mind works	1	2	3	4	5	6
16. I frequently take time to reflect on my thoughts	1	2	3	4	5	6
17. Often I find it difficult to make sense of the way I feel about things (R)	1	2	3	4	5	6
18. It is important to me to be able to understand how my thoughts arise	1	2	3	4	5	6
19. I often think about the way I feel about things	1	2	3	4	5	6
20. I usually know why I feel the way I do	1	2	3	4	5	6

Self-Compassion Scale

HOW I TYPICALLY ACT TOWARDS MYSELF IN DIFFICULT TIMES

Please read each statement carefully before answering. To the left of each item, indicate how often you behave in the stated manner, using the following scale:

STATEMENTS	Almost Never				Almost Always
1. I'm disapproving and judgmental about my own flaws and inadequacies.	1	2	3	4	5
2. When I'm feeling down I tend to obsess and fixate on everything that's wrong.	1	2	3	4	5
3. When things are going badly for me, I see the difficulties as part of life that everyone goes through.	1	2	3	4	5
4. When I think about my inadequacies, it tends to make me feel more separate and cut off from the rest of the world.	1	2	3	4	5
5. I try to be loving towards myself when I'm feeling emotional pain.	1	2	3	4	5
6. When I fail at something important to me I become consumed by feelings of inadequacy.	1	2	3	4	5
7. When I'm down and out, I remind myself that there are lots of other people in the world feeling like I am.	1	2	3	4	5
8. When times are really difficult, I tend to be tough on myself.	1	2	3	4	5
9. When something upsets me I try to keep my emotions in balance.	1	2	3	4	5
10. When I feel inadequate in some way, I try to remind myself that feelings of inadequacy are shared by most people.	1	2	3	4	5
11. I'm intolerant and impatient towards those aspects of my personality I don't like.	1	2	3	4	5
12. When I'm going through a very hard time, I give myself the caring and tenderness I need.	1	2	3	4	5
13. When I'm feeling down, I tend to feel like most other people are probably happier than I am.	1	2	3	4	5
14. When something painful happens I try to take a balanced view of the situation.	1	2	3	4	5
15. I try to see my failings as part of the human condition.	1	2	3	4	5
16. When I see aspects of myself that I don't like, I get down on myself.	1	2	3	4	5

STATEMENTS	Almost Never				Almost Always
17. When I fail at something important to me I try to keep things in perspective.	1	2	3	4	5
18. When I’m really struggling, I tend to feel like other people must be having an easier time of it.	1	2	3	4	5
19. I’m kind to myself when I’m experiencing suffering.	1	2	3	4	5
20. When something upsets me I get carried away with my feelings.	1	2	3	4	5
21. I can be a bit cold-hearted towards myself when I'm experiencing suffering.	1	2	3	4	5
22. When I'm feeling down I try to approach my feelings with curiosity and openness.	1	2	3	4	5
23. I’m tolerant of my own flaws and inadequacies.	1	2	3	4	5
24. When something painful happens I tend to blow the incident out of proportion.	1	2	3	4	5
25. When I fail at something that's important to me, I tend to feel alone in my failure.	1	2	3	4	5
26. I try to be understanding and patient towards those aspects of my personality I don't like.	1	2	3	4	5

Professional Quality of Life Scale

When you *[help]* people you have direct contact with their lives. As you may have found, your compassion for those you *[help]* can affect you in positive and negative ways. Below are some questions about your experiences, both positive and negative, as a *[helper]*. Consider each of the following questions about you and your current work situation. Select the number that honestly reflects how frequently you experienced these things in the last 30 days.

STATEMENTS	Never	Rarely	Sometimes	Often	Very Often
1. I am happy.	1	2	3	4	5
2. I am preoccupied with more than one person I *[help]*.	1	2	3	4	5
3. I get satisfaction from being able to *[help]* people.	1	2	3	4	5
4. I feel connected to others.	1	2	3	4	5
5. I jump or am startled by unexpected sounds	1	2	3	4	5
6. I feel invigorated after working with those I *[help]*.	1	2	3	4	5
7. I find it difficult to separate my personal life from my life as a *[helper]*.	1	2	3	4	5
8. I am not as productive at work because I am losing sleep over traumatic experiences of a person I *[help]*.	1	2	3	4	5
9. I think that I might have been affected by the traumatic stress of those I *[help]*.	1	2	3	4	5
10. I feel trapped by my job as a *[helper]*.	1	2	3	4	5
11. Because of my *[helping]*, I have felt “on edge” about various things.	1	2	3	4	5
12. I like my work as a *[helper]*.	1	2	3	4	5
13. I feel depressed because of the traumatic experiences of the people I *[help]*.	1	2	3	4	5
14. I feel as though I am experiencing the trauma of someone I have *[helped]*.	1	2	3	4	5
15. I have beliefs that sustain me.	1	2	3	4	5
16. I am pleased with how I am able to keep up with *[helping]* techniques and protocols.	1	2	3	4	5
17. I am the person I always wanted to be.	1	2	3	4	5
18. My work makes me feel satisfied.	1	2	3	4	5

19. I feel worn out because of my work as a *[helper]*.	**1**	**2**	**3**	**4**	**5**
20. I have happy thoughts and feelings about those I *[help]* and how I could help them.	**1**	**2**	**3**	**4**	**5**
21. I feel overwhelmed because my case [work] load seems endless.	**1**	**2**	**3**	**4**	**5**
22. I believe I can make a difference through my work.	**1**	**2**	**3**	**4**	**5**
23. I avoid certain activities or situations because they remind me of frightening experiences of the people I *[help]*.	**1**	**2**	**3**	**4**	**5**
24. I am proud of what I can do to *[help]*.	**1**	**2**	**3**	**4**	**5**
25. As a result of my *[helping]*, I have intrusive, frightening thoughts.	**1**	**2**	**3**	**4**	**5**
26. I feel "bogged down" by the system.	**1**	**2**	**3**	**4**	**5**
27. I have thoughts that I am a "success" as a *[helper]*.	**1**	**2**	**3**	**4**	**5**
28. I can't recall important parts of my work with trauma victims.	**1**	**2**	**3**	**4**	**5**
29. I am a very caring person.	**1**	**2**	**3**	**4**	**5**
30. I am happy that I chose to do this work.	**1**	**2**	**3**	**4**	**5**
