# Supplementary material for: Modifiable Protective Strategies and System-Level Predictors of Professional Quality of Life in Oncology Nurses: A Secondary Analysis
Source: SAGE Open Nurs. 2025 Dec 22;11:23779608251407811. doi: 10.1177/23779608251407811 (PMC12722642; doi:10.1177/23779608251407811)
Supplement: sj-docx-2-son-10.1177_23779608251407811 - Supplemental material for Modifiable Protective Strategies and System-Level Predictors of Professional Quality of Life in Oncology Nurses: A Secondary Analysis [file sj-docx-2-son-10.1177_23779608251407811.docx]

**COREQ Checklist**

| **Domain** | **Item No.** | **Item** | **Description / Guide Question** | **Response** |
| --- | --- | --- | --- | --- |
| **Domain 1: Research team and reflexivity** |  |  |  |  |
| **Personal characteristics** | 1 | Interviewer/facilitator | Which author(s) conducted the interview or focus group? | NA |
|  | 2 | Credentials | What were the researcher’s credentials? (e.g., PhD, MD) | Yes |
|  | 3 | Occupation | What was their occupation at the time of the study? | Yes |
|  | 4 | Gender | Was the researcher male or female? | Yes |
|  | 5 | Experience and training | What experience or training did the researcher have? | NA |
| **Relationship with participants** | 6 | Relationship established | Was a relationship established prior to study commencement? | Yes |
|  | 7 | Participant knowledge of the interviewer | What did participants know about the researcher (e.g., goals, reasons for doing the research)? | Goals & reason for research |
|  | 8 | Interviewer characteristics | What characteristics were reported about the interviewer/facilitator (e.g., bias, assumptions, reasons for interest)? | NA |
| **Domain 2: Study design** |  |  |  |  |
| **Theoretical framework** | 9 | Methodological orientation and theory | What methodological orientation was stated to underpin the study (e.g., grounded theory, discourse analysis, ethnography, phenomenology, content analysis)? | Yes |
| **Participant selection** | 10 | Sampling | How were participants selected? (e.g., purposive, convenience, snowball) | Yes |
|  | 11 | Method of approach | How were participants approached? (e.g., face-to-face, telephone, mail, email) | Yes |
|  | 12 | Sample size | How many participants were in the study? | Yes |
|  | 13 | Non-participation | How many people refused to participate or dropped out? Reasons? | Yes |
| **Setting** | 14 | Setting of data collection | Where was the data collected? (e.g., home, clinic, workplace) | Yes |
|  | 15 | Presence of non-participants | Was anyone else present besides participants and researchers? | NA |
|  | 16 | Description of sample | What are the important characteristics of the sample (e.g., demographic data, date)? | Yes |
| **Data collection** | 17 | Interview guide | Were questions, prompts, or guides provided by the authors? Was it pilot tested? | Yes |
|  | 18 | Repeat interviews | Were repeat interviews carried out? If yes, how many? | NA |
|  | 19 | Audio/visual recording | Did the research use audio or visual recording to collect the data? | NA |
|  | 20 | Field notes | Were field notes made during and/or after the interview or focus group? | NA |
|  | 21 | Duration | What was the duration of the interviews or focus groups? | NA |
|  | 22 | Data saturation | Was data saturation discussed? | NA |
|  | 23 | Transcripts returned | Were transcripts returned to participants for comment and/or correction? | No |
| **Domain 3: Analysis and findings** |  |  |  |  |
| **Data analysis** | 24 | Number of data coders | How many data coders coded the data? | 2 |
|  | 25 | Description of the coding tree | Did authors provide a description of the coding tree? | No |
|  | 26 | Derivation of themes | Were themes identified in advance or derived from the data? | Advanced |
|  | 27 | Software | What software, if applicable, was used to manage the data? | No |
|  | 28 | Participant checking | Did participants provide feedback on the findings? | No |
| **Reporting** | 29 | Quotations presented | Were participant quotations presented to illustrate the themes/findings? Were they identified (e.g., participant number)? | Yes |
|  | 30 | Data and findings consistent | Was there consistency between the data presented and the findings? | Yes |
|  | 31 | Clarity of major themes | Were major themes clearly presented in the findings? | Yes |
|  | 32 | Clarity of minor themes | Is there a description of diverse cases or discussion of minor themes? | Yes |
